# Masseter muscle change during chemoradiotherapy for esophageal cancer: survival and immunonutritional associations compared with the psoas muscle index

**DOI:** 10.1007/s00595-026-03288-y

**Published:** 2026-04-24

**Authors:** Tenshi Makiyama, Yasunori Matsumoto, Takeshi Toyozumi, Akira Nakano, Nobufumi Sekino, Tadashi Shiraishi, Koichiro Okada, Masanari Yamada, Yuri Nishioka, Akane Morimoto, Michihiro Maruyama

**Affiliations:** Department of Frontier Surgery, Graduate School of Medicine, Chiba University, Chiba, Japan

**Keywords:** Esophageal squamous cell carcinoma, Masseter muscle, Nutrition assessment, Prognosis, Radiotherapy

## Abstract

**Purpose:**

Treatment-related muscle loss is common in esophageal squamous cell carcinoma (ESCC). The masseter reflects chewing–swallowing musculature; therefore, we evaluated whether preserving the masseter muscle index (ΔMMI) during preoperative chemoradiotherapy (CRT) relates more to survival and immunonutritional change, than preserving the psoas muscle index (ΔPMI).

**Methods:**

The subjects of this retrospective analysis were 78 ESCC patients who underwent CRT followed by esophagectomy. MMI and PMI were measured on contrast-enhanced CT at baseline (TP0) and post-CRT (TP1). Immunonutritional markers were assessed at TP0–TP2 (3 months postoperatively, ± 14 days) based on percent change (Δ). Associations with interval changes were tested with ANCOVA, adjusted for baseline, age, and sex. Overall survival (OS) and recurrence-free survival (RFS) were evaluated by Kaplan–Meier using median-split Δ over TP0→TP1.

**Results:**

Better maintenance of ΔMMI during CRT was related to better OS (borderline) and favorable profiles; namely, improved BMI maintenance and lower Glasgow Prognostic Score (GPS), over TP0→TP1. In contrast, greater ΔPMI loss did not separate OS, but was associated with worse RFS and consistent deterioration in the indices of BMI, geriatric nutritional risk index (GNRI), and albumin, through TP0→TP2.

**Conclusions:**

Preserving ΔMMI during CRT relates to survival and immunonutritional stability, complementing ΔPMI as a systemic marker of decline. Incorporating masseter imaging into routine assessment and testing MMI-guided perioperative support warrants prospective evaluation.

**Supplementary Information:**

The online version contains supplementary material available at 10.1007/s00595-026-03288-y.

## Introduction

Esophageal cancer remains a major global health burden, ranking the 11th most prevalent cancer and the 7th most lethal cancer worldwide [1]. In esophageal squamous cell carcinoma (ESCC), host-related factors, particularly nutritional status and sarcopenia, are being increasingly recognized as determinants of postoperative complications and survival [2, 3]. Neoadjuvant treatment can exacerbate skeletal muscle depletion, and meta-analytic evidence links treatment-related muscle loss with the poor prognosis of gastrointestinal malignancies [4]. For example, objective nutritional indices such as the Prognostic Nutritional Index (PNI) show independent associations with the survival of ESCC patients, underscoring the prognostic role of preoperative nutritional status [5]. A recent review synthesized evidence on serum tumor markers in esophageal and gastric cancers, underscoring their adjunctive value for prognosis and treatment monitoring [6].

Traditionally, skeletal muscle assessment has relied on trunk-based indices such as the lumbar skeletal muscle index (L3 SMI) or the psoas muscle index (PMI), measured on abdominal computed tomography (CT) [7, 8]. Quantification of the skeletal muscle cross-sectional area at the third lumbar vertebra (L3) on routine CT has long served as a pragmatic standard in oncologic body composition research [9]. However, these trunk-based indices may not fully capture the oropharyngeal musculature, which is directly exposed to chemoradiotherapy (CRT)-related toxicities such as mucositis, odynophagia, and reduced oral intake. The masseter muscle index (MMI) quantifies chewing and swallowing musculature and may reflect a distinct, treatment-sensitive dimension of vulnerability related to dysphagia, aspiration, and nutrition. Sarcopenic dysphagia, characterized by loss of swallowing-related muscle mass and function, has been implicated in aspiration and poor outcomes [10]. In ESCC, CRT often worsens mucositis and odynophagia, plausibly reducing oral intake and selectively causing atrophy of the masseter muscle. Previous studies have reported associations between low preoperative MMI and postoperative pneumonia and mortality after esophagectomy [11, 12]; however, most of these studies examined static measurements rather than dynamic changes.

Postoperative pneumonia is a major determinant of long-term outcomes after esophagectomy, with several studies linking early pulmonary complications to poorer overall and recurrence-free survival [13, 14]. Given CRT-related eating difficulties, a loss of chewing and swallowing musculature could plausibly increase pneumonia risk through dysphagia and impaired airway protection. Therefore, beyond trunk indices, evaluating peri-treatment changes in the MMI may provide complementary prognostic insight. We aimed to establish whether changes in the MMI (ΔMMI from initial presentation to post-CRT, preoperative assessment) reflect treatment-related vulnerability and are associated with postoperative complications and survival. In this study, timepoints are defined as TP0 (baseline), TP1 (after completion of CRT and preoperative), and TP2 (3 months postoperatively, ± 14 days). We quantified MMI and PMI on CT at TP0 and TP1 and examined their associations with immunonutritional and inflammatory markers assessed at TP0, TP1, and TP2, postoperative pneumonia, and long-term outcomes in a single-center cohort of patients with ESCC, who underwent surgery after CRT (neoadjuvant or salvage).

## Methods

### Study design and patient selection

This was a single-center, retrospective observational study. We screened 316 consecutive patients with pathologically confirmed ESCC, who underwent curative-intent esophagectomy at Chiba University Hospital between January, 2011 and August, 2020. Curative intent was defined as resection for non-metastatic disease aiming at R0 resection. Staging followed the Union for International Cancer Control (UICC) 8th edition [15], and perioperative management conformed to the Japan Esophageal Society guidelines [16].

For this analysis, eligibility required completion of preoperative CRT and measurable MMI (cm²/m²) and PMI (cm²/m²) on contrast-enhanced CT, specifically at TP0 (baseline) and TP1 (post-CRT). We restricted the analytic cohort to patients who underwent curative-intent esophagectomy after CRT (neoadjuvant or salvage) because the primary endpoints (OS and RFS) were defined from the date of esophagectomy. Patients treated with definitive CRT without subsequent esophagectomy were not included in the analysis, as the treatment intent and appropriate time-to-event definitions differ substantially and would introduce substantial selection and comparability bias. We also excluded patients who did not undergo curative surgery, lacked any of the required CT scans, or had either MMI or PMI that could not be measured. Clinical and laboratory data were required at TP0, TP1, and TP2. Analyses at TP2 required further complete data at that timepoint. After applying these criteria, 78 patients comprised the analytic cohort. All 78 were included in the TP0 and TP1 analyses, and 76 were included in the TP2 analyses, after excluding two deaths within 3 months postoperatively.

Representative CRT regimens included cisplatin plus 5-fluorouracil. The cohort comprised patients who underwent esophagectomy after CRT, including neoadjuvant CRT (typically 40 Gy in 20 fractions) and salvage esophagectomy following definitive CRT (typically 60 Gy in 30 fractions). All esophagectomies were performed via a thoracoabdominal approach. Postoperative complications within 30 days were graded using the Clavien–Dindo classification, and postoperative pneumonia was defined as grade ≥ II [17]. Baseline swallowing function and oral intake status before CRT were assessed using the Mellow–Pinkas dysphagia score at TP0 (baseline), which grades dysphagia according to the maximum diet consistency tolerated (0–4, with higher scores indicating more severe dysphagia) [18]. Tumor location was categorized according to the UICC criteria as cervical, upper, middle, or lower esophagus [15].

This study was approved by the institutional review board of Chiba University Hospital (Approval No. HK202409-13). All procedures adhered to the ethical standards established by the responsible committee on human experimentation, as well as the Helsinki Declaration of 1964 and its subsequent amendments. Patient consent was obtained using an opt-out approach, ensuring participants were informed about the research and given the opportunity to withdraw their data if they chose to.

### CT acquisition and muscle indices

On axial CT, bilateral masseter muscles were measured at the inferior border of the maxilla, and bilateral psoas muscles were measured at L3. Areas (cm²) were traced using SYNAPSE VINCENT (FUJIFILM, Tokyo, Japan), summed bilaterally, and normalized to a height squared to yield indices (cm²/m²): MMI and PMI. Percent change (Δ) over an interval a→b was defined as (([value at b − value at a] / value at a) × 100), with negative values indicating loss. The prespecified imaging interval for muscle indices was TP0→TP1. TP2 was used only for immunonutritional endpoints, not for CT-based muscle measurements.

All measurements were performed by a single evaluator blinded to clinical outcomes (OS/RFS) and postoperative complications, using a prespecified protocol with fixed anatomical landmarks to minimize measurement variability.

### Immunonutritional and inflammatory variables

Age and sex were recorded at baseline (TP0). Body mass index (BMI) and laboratory measures (albumin, C-reactive protein [CRP], total cholesterol, neutrophil, lymphocyte, and monocyte counts, and platelets) were obtained at TP0, TP1, and TP2. Derived indices included the PNI (PNI = 10 × albumin + 0.005 × lymphocytes/µL), geriatric nutritional risk index (GNRI = 14.89 × albumin + 41.7 × [weight/ideal weight], with weight/ideal weight capped at 1.0; ideal weight = 22 × height²), C-reactive protein/albumin ratio (CAR = CRP/albumin), neutrophil-to-lymphocyte ratio (NLR), platelet-to-lymphocyte ratio (PLR), lymphocyte-to-monocyte ratio (LMR), and systemic immune-inflammation index (SII = neutrophils × platelets/lymphocytes). The Controlling Nutritional Status (CONUT) score (0–12) [19] and Glasgow Prognostic Score (GPS: 0–2) [20] were calculated using standard criteria.

### Statistical analyses

Continuous variables are summarized as median (interquartile range [IQR]) and categorical variables as counts. For two-group comparisons, continuous variables were tested using the two-sided Mann–Whitney U test (Wilcoxon rank-sum). Categorical variables were compared using Fisher’s exact test for 2 × 2 tables and Pearson’s chi-square test for tables with > 2 categories such as tumor location or clinical/pathological stage.

Nutritional and inflammatory changes were computed over TP0→TP1, TP1→TP2, and TP0→TP2 as absolute changes (ΔZ=Zend−Zstart) for the prespecified panel (BMI, albumin, platelet count, CRP, PNI, GNRI, CONUT, GPS, CAR, NLR, PLR, LMR, and SII). CONUT and GPS were analyzed as continuous numeric variables using their integer score values (CONUT 0–12; GPS 0–2). Because the exposures were within-person percentage changes (ΔMMI%, ΔPMI%), which were less sensitive to baseline sex-based differences in absolute muscle size, and the cohort was predominantly male, we did not stratify by sex. Instead, sex was included as an a priori covariate in adjusted analyses.

Monotonic associations were examined using adjusted change models (analysis of covariance [ANCOVA]) rather than unadjusted tests. Specifically, for each marker Z and interval, ΔZ was regressed on the exposure and adjusted for the interval’s starting value of Z (TP0 for TP0→TP1/TP0→TP2; TP1 for TP1→TP2), age (years at TP0), and sex. Exposures were analyzed in two ways: (i) as continuous ΔMMI or ΔPMI (fitted in separate models to avoid collinearity, with effect sizes reported as slopes β₁ per 1% change in ΔMMI or ΔPMI, alongside 95% confidence intervals [CIs] and P values), and (ii) dichotomized at the cohort median (greater loss = Δ ≤ median vs. lesser loss = Δ > median).

Accordingly, adjusted change models took the form:

$$\Delta {\rm{Z}}\, = \,\alpha \, + \, {\beta_0} \cdot{\rm{Zstart}}\, + \, {\beta_1} \cdot{\rm{Exposure}}\, + \, {\beta_2} \cdot{\rm{Age}}\, + \, {\beta_3} \cdot{\rm{Sex}}\, + \,\varepsilon$$

Here, Zstart is the interval’s starting value of Z (TP0 for TP0→TP1 and TP0→TP2; TP1 for TP1→TP2). Exposure was specified as either the continuous percentage change (ΔMMI% or ΔPMI%, fit in separate models to avoid collinearity) or as a binary indicator defined by the cohort median of Δ over TP0→TP1 (greater loss = Δ ≤ median; lesser loss = Δ > median). For continuous exposures, β₁ is the slope representing the change in ΔZ per 1% change in ΔMMI% (or ΔPMI%), reported with 95% confidence intervals (CIs) and P values. For dichotomized exposures, β₁ corresponds to the adjusted least-squares mean (LSMean) difference in ΔZ computed as greater loss (Δ ≤ median) − lesser loss (Δ > median), with 95% CIs. β₂ is the slope per 1-year increase in age. β₃ is the adjusted mean difference in ΔZ between sex categories (0 = male, 1 = female; reference = male). α denotes the intercept and ε the residual error term.

Overall survival (OS) and recurrence-free survival (RFS) were analyzed using Kaplan–Meier curves and log-rank tests. OS was defined as the time from esophagectomy to death from any cause, with surviving patients censored at last follow-up. RFS was defined as the time from esophagectomy to the first documented recurrence or death, whichever occurred first. Primary stratifications were the median splits of ΔMMI% and ΔPMI% (TP0→TP1), and 60-month survival proportions were obtained from Kaplan–Meier estimates.

Additional contrasts considered postoperative pneumonia status. As an exploratory analysis, we also performed a four-group stratification, defined by the combination of the median splits of ΔMMI% and ΔPMI% (2 × 2) and compared OS and RFS across the four groups using log-rank tests. As a sensitivity analysis, Cox proportional hazards models were fitted to estimate hazard ratios for ΔMMI strata, with the greater-loss group as the reference category. These models were additionally adjusted for baseline Mellow–Pinkas dysphagia score (TP0) and dichotomized tumor location (cervical/upper vs. middle/lower).

Analyses used complete cases per endpoint, with no imputation. All tests were two-sided (α = 0.05), no multiplicity adjustment was applied, and estimates are reported with 95% CIs alongside P values. All analyses were performed using JMP Pro 17 (SAS Institute Inc., Cary, NC, USA).

## Results

### Patient characteristics

Table 1 summarizes the baseline characteristics. A total of 78 patients met the eligibility criteria (median age: 69.5 years [IQR: 65.0–73]; 67/78 men, 85.9%). Using a cohort-median split (greater loss ≤ − 6.98% vs. lesser loss), baseline profiles were broadly similar: age and sex did not differ (both *P* > 0.3). Radiotherapy dose (40 vs. 60 Gy) and stage distribution showed no material imbalances between strata. Tumor location and baseline swallowing function (Mellow–Pinkas dysphagia score at TP0) were also well balanced between the strata (*P* = 0.1380 and *P* = 0.5230, respectively).

Postoperative pneumonia developed in 21/78 patients (26.9%) and was similarly distributed between the ΔMMI strata (greater loss 12/39 [30.8%] vs. lesser loss 9/39 [23.1%]; *P* = 0.6103).

**Table 1:** Patient characteristics and computed tomography-derived muscle indices (TP0–TP1), stratified by the masseter muscle index (ΔMMI)

Variable	ALL *n*=78	ΔMMI Greater Loss (≤ median) *n*=39	ΔMMI Lesser Loss (> median) *n*=39	*P* value
Age (years)	69.5 (65 – 73)	70 (66 – 72)	69 (61 – 74)	0.3341
Sex (Male / Female)	67/11	35/4	32/7	0.5170
MMI(TP0) (cm^2^/m^2^)	3.671 (2.981 – 4.162)	3.812 (3.358 – 4.155)	3.522 (2.801 – 4.212)	0.1121
MMI(TP1) (cm^2^/m^2^)	3.314 (2.907 – 4.130)	3.186 (2.789 – 3.566)	3.688 (2.918 – 4.296)	0.0753
PMI(TP0) (cm^2^/m^2^)	4.883 (4.125 – 6.274)	5.360 (4.328 – 6.394)	4.788 (4.121 – 5.860)	0.2304
PMI(TP1) (cm^2^/m^2^)	4.866 (4.105 – 5.710)	4.878 (4.087 – 5.796)	4.713 (4.111 – 5.513)	0.9125
ΔMMI(TP0→TP1)(%)	–6.98 (–13.91 to 0.49)	–13.80 (–19.54 to –10.46)	0.46 (–3.98 to 13.34)	<0.0001
ΔPMI(TP0→TP1)(%)	–4.70 (–13.07 to 3.77)	–7.04 (–14.40 to 1.25)	–3.21 (–11.07 to 5.94)	0.0838
Postoperative pneumonia	21	12	9	0.6103
Tumor location (Cervical/Upper/Middle/Lower)	6/10/37/25	1/3/20/15	5/7/17/10	0.1380
Dysphagia score (0/1/2/3/4)	25/21/23/9/0	10/10/14/5/0	15/11/9/4/0	0.5230
Radiotherapy dose (40/60 Gy)	63/15	32/7	31/8	1.0000
cT(0/1/2/3/4a/4b)	0/3/2/23/16/34	0/1/2/9/7/20	0/2/0/14/9/14	0.3162
cN(0/1/2/3)	4/20/25/29	2/14/9/14	2/6/16/15	0.1581
cM(0/1)	78/0	39/0	39/0	N/A
cStage(0/1/2/3/4a/4b)	0/0/1/23/54/0	0/0/0/12/27/0	0/0/1/11/27/0	0.5935
pT(Tis/1a/1b/2/3/4a/4b)	22/5/5/10/34/2/0	9/2/3/4/20/1/0	13/3/2/6/14/1/0	0.7635
pN(0/1/2/3)	40/15/19/4	20/7/10/2	20/8/9/2	0.9894
pM(0/1)	78/0	39/0	39/0	N/A
pStage(0/1/2a/2b/3a/3b/4a/4b)	22/1/7/13/4/26/5/0	9/1/3/8/1/14/3/0	13/0/4/5/3/12/2/0	0.6880

Data are shown as the median (IQR) or n values, as appropriate. P values were calculated using the Mann–Whitney U test for continuous variables; Fisher’s exact test for 2 × 2 categorical variables; and Pearson’s chi-square test for categorical variables with more than two categories. P values were reported as N/A when not applicable. Δ denotes percent change; ΔMMI and ΔPMI were computed as ([TP1 − TP0] / TP0 × 100). TP0, baseline (pre-CRT); TP1, post-CRT, preoperative assessment. Staging follows UICC 8th edition. Radiation dose is expressed in Gray (Gy). Abbreviations: CRT, chemoradiotherapy; MMI, masseter muscle index; PMI, psoas muscle index; Gy, Gray; N/A, not applicable

### Absolute muscle indices and interval changes during CRT (TP0→TP1)

**Fig. 1:**
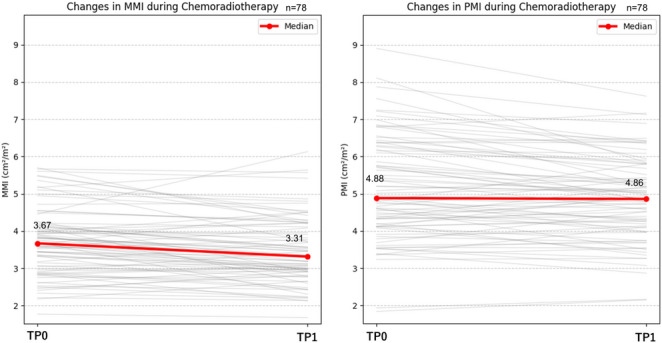
Changes in masseter and psoas muscle indices during chemoradiotherapy. Paired-line plots of the masseter muscle index (MMI, left) and psoas muscle index (PMI, right) at TP0 and TP1 (*n* = 78). Light gray lines indicate individual patient trajectories, and red lines indicate the cohort medians at each time point (median values are displayed). Abbreviations: MMI, masseter muscle index; PMI, psoas muscle index; TP0,baseline (pre-CRT); TP1, post-CRT (preoperative)

Figure 1 shows the paired-line plots of absolute MMI and PMI at TP0 and TP1 (per-patient trajectories). Table 1 also shows the baseline (TP0) cohort-level MMI and PMI. The median absolute values (cm²/m²) were as follows: MMI—TP0: 3.67 (2.98–4.16) → TP1: 3.31 (2.90–4.13); PMI—TP0: 4.88 (4.12–6.27) → TP1: 4.86 (4.10–5.71). The median percent changes during CRT were: TP0→TP1—ΔMMI − 6.98% (IQR − 13.91 to 0.49) and ΔPMI − 4.70% (IQR − 13.07 to 3.77). Although cohort-level PMI medians at TP0 and TP1 were similar, the paired median ΔPMI indicated a modest within-patient decline.

### Immunonutritional and inflammatory profiles across TP0, TP1, and TP2

Table 2 summarizes the main immunonutritional and inflammatory indices across TP0, TP1, and TP2. Supplementary Table S2 shows the additional hematologic immune–inflammation indices: platelet count, NLR, PLR, LMR, SII, and CONUT. Cohort-wide, median immunonutritional indices declined during CRT and remained low at 3 months: BMI: 22.0→20.9→19.0; albumin: 4.1→3.7→3.6 g/dL; PNI: 50.3→40.5→40.5; GNRI: 101.3→92.5→89.9. GPS (≥ 1) showed only modest fluctuations over time (20.5%→15.4%→18.4%) and did not exceed the baseline level.

When stratified by early masseter loss (ΔMMI TP0→TP1, median split), baseline profiles were broadly similar (Table 2). A few isolated between-group differences were observed at TP0 and TP1, whereas no between-group differences reached significance at TP2.

**Table 2:** Immunonutritional and inflammatory indices stratified by the masseter muscle index (ΔMMI) (TP0→TP1) median

Variable	All patients	ΔMMI Greater Loss (≤ median) *n*=39	ΔMMI Lesser Loss (> median) *n*=39	*P* value
**TP0** **(baseline)** **(*n*=78)**				
BMI (kg/m²)	22.0 (19.6–24.1)	23.0 (19.5–24.6)	21.2 (19.2–23.2)	0.2044
Albumin (g/dL)	4.1 (3.9–4.3)	4.2 (4.0–4.3)	4.1 (3.8–4.3)	0.2988
PNI	50.3 (47.8–53.7)	51.4 (48.9–54.1)	48.8 (45.9–52.3)	0.0245
GNRI	101.3 (95.6–105.7)	101.3 (96.9–104.2)	101.3 (94.9–105.7)	0.4180
CRP (mg/dL)	0.30 (0.10–0.80)	0.30 (0.10–0.85)	0.30 (0.10–0.65)	0.9960
CAR	0.069 (0.024–0.203)	0.067 (0.026–0.210)	0.081 (0.023–0.162)	1.0000
GPS (0/1/2)	62/12/4	30/9/0	32/3/4	0.0292
**TP1** **(post-CRT,** **preoperative)** **(*n*=78)**		**ΔMMI** **Greater** **Loss** **(≤ median)** ***n*=39**	**ΔMMI** **Lesser** **Loss** **(> median)** ***n*=39**	**P value**
BMI (kg/m²)	20.9 (19.1–22.9)	20.8 (19.4–22.3)	21.1 (18.5–23.1)	0.9442
Albumin (g/dL)	3.7 (3.4–3.9)	3.8 (3.3–3.9)	3.7 (3.4–3.9)	0.5913
PNI	40.5 (38.0–44.1)	41.3 (38.0–44.3)	40.3 (38.3–43.4)	0.8730
GNRI	92.5 (88.1–98.3)	93.8 (87.7–99.0)	91.3 (89.1–96.8)	0.4505
CRP (mg/dL)	0.20 (0.10–0.65)	0.16 (0.07–0.47)	0.40 (0.10–0.96)	0.0354
CAR	0.060 (0.025–0.178)	0.044 (0.018–0.120)	0.093 (0.029–0.270)	0.0349
GPS (0/1/2)	66/3/9	29/3/7	37/0/2	0.0343
**TP2** **(3 months postoperatively)** **(*n*=76)**		**ΔMMI** **Greater** **Loss** **(≤ median)** ***n*=38**	**ΔMMI** **Lesser** **Loss** **(> median)** ***n*=38**	**P value**
BMI (kg/m²)	19.0 (17.8–20.8)	19.0 (17.9–20.4)	19.3 (17.8–21.1)	0.6664
Albumin (g/dL)	3.6 (3.2–3.8)	3.7 (3.3–4.0)	3.6 (3.1–3.7)	0.1503
PNI	40.5 (35.7–43.4)	40.9 (35.7–43.7)	39.8 (35.8–42.5)	0.3366
GNRI	89.9 (82.0–94.9)	91.3 (84.2–94.6)	88.7 (80.3–94.8)	0.3800
CRP (mg/dL)	0.20 (0.10–0.70)	0.17 (0.10–0.70)	0.24 (0.12–0.65)	0.3549
CAR	0.062 (0.026–0.194)	0.045 (0.024–0.193)	0.067 (0.035–0.189)	0.3209
GPS (0/1/2)	62/3/11	30/1/7	32/2/4	0.5444

Data are presented as median (interquartile range [IQR]) or n values, as appropriate. P values compare ΔMMI strata (ΔMMI ≤ cohort median vs. ΔMMI > cohort median) at each time point and were calculated using the Mann–Whitney U test for continuous variables; Fisher’s exact test for 2 × 2 categorical variables; and Pearson’s chi-square test for categorical variables with more than two categories (including the full score distribution of GPS). ΔMMI is the percent change in MMI during CRT (TP0→TP1), defined as ([TP1−TP0]/TP0)×100; negative values indicate loss (cohort median ΔMMI = −6.98%). TP0, baseline (pre-CRT); TP1, post-CRT, preoperative assessment; TP2, 3 months postoperatively (±14 days). Denominators may vary because of missing values, as shown. Abbreviations: BMI, body mass index; CAR, C-reactive protein/albumin ratio; CRP, C-reactive protein; CRT, chemoradiotherapy; GNRI, geriatric nutritional risk index; GPS, Glasgow Prognostic Score; MMI, masseter muscle index; PNI, prognostic nutritional index

### Survival analysis

**Fig. 2:**
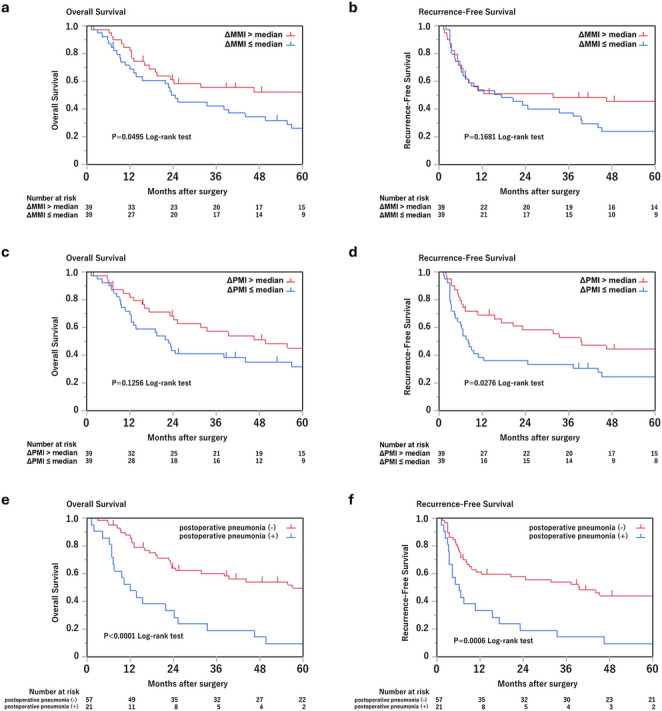
Kaplan–Meier survival by early muscle change (ΔMMI/ΔPMI, TP0→TP1) and postoperative pneumonia (a, b) Overall survival (OS) and recurrence-free survival (RFS) stratified by ΔMMI using a cohort-median split. (c, d) OS and RFS stratified by ΔPMI using a cohort-median split. (e, f) OS and RFS stratified by postoperative pneumonia (− vs +; definition in Methods). Percent change (Δ) was calculated as (TP1 − TP0)/ TP0 × 100. For ΔMMI and ΔPMI, values > median indicate lesser loss (higher Δ), whereas values ≤ median indicate greater loss (ties included). Median cutoffs were −6.98% for ΔMMI and −4.70% for ΔPMI. Tick marks indicate censoring, and numbers at risk are shown below each plot. Groups were compared using the log-rank test (P values shown). Abbreviations: MMI, masseter muscle index; PMI, psoas muscle index; OS, overall survival; RFS, recurrence-free survival; TP0, baseline (pre-CRT); TP1, post-CRT (preoperative)

Figure 2 shows the Kaplan–Meier curves (A–B: ΔMMI vs. OS/RFS; C–D: ΔPMI vs. OS/RFS; E–F: postoperative pneumonia vs. OS/RFS). After 60 months, the cohort-level OS and RFS rates were 38.3% and 34.5%, respectively. Stratification by the cohort median of ΔMMI (TP0→TP1) (greater loss ≤ − 6.98% vs. lesser loss) yielded a borderline-significant OS difference: 25.9% vs. 52.1% (log-rank *P* = 0.0495). Although the ΔMMI and ΔPMI curves may appear visually similar, the survival separation differed by endpoint in this cohort. In a sensitivity Cox model additionally adjusting for baseline Mellow–Pinkas dysphagia score (TP0) and dichotomized tumor location (cervical/upper vs. middle/lower), the lesser-loss ΔMMI group remained associated with improved OS compared with the greater-loss group (reference) (HR 0.49, 95% CI 0.25–0.92; *P* = 0.0307) (Supplementary Table S1). In contrast, a parallel split by ΔPMI (TP0→TP1) (greater loss ≤ − 4.70% vs. lesser loss) did not separate OS (31.5% vs. 45.2%, *P* = 0.1256). The pattern was reversed for RFS: ΔPMI (TP0→TP1) separated RFS (24.6% vs. 44.2%, *P* = 0.0276), whereas ΔMMI (TP0→TP1) did not (24.0% vs. 45.5%, *P* = 0.1681).

Postoperative pneumonia provided important context. Pneumonia developed within 30 days in 21/78 patients and was associated with significantly worse outcomes: 5-year OS (9.5% vs. 48.6%) (*P* < 0.0001) and 5-year RFS (9.5% vs. 44.1%) (*P* = 0.0006).

As an exploratory analysis, patients were further stratified into four groups using the cohort medians of ΔMMI (− 6.98%) and ΔPMI (− 4.70%) (2 × 2). Overall differences were borderline for OS (log-rank *P* = 0.0523) and significant for RFS (*P* = 0.0381) (Supplementary Fig. S1). Across both endpoints, the ΔMMI ≤ median/ΔPMI ≤ median group showed the poorest outcomes.

### Association between ΔMMI/ΔPMI and changes in nutritional/inflammatory endpoints (ANCOVA)

**Fig. 3:**
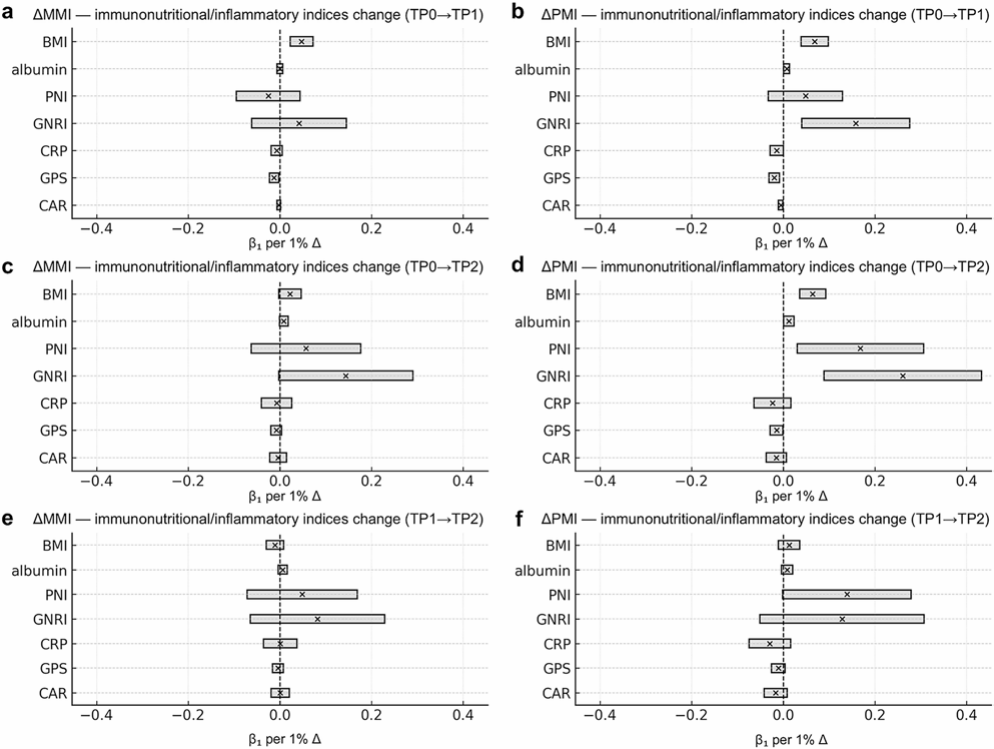
Associations between ΔMMI/ΔPMI and changes in immunonutritional/inflammatory indices across intervals (ANCOVA) (a, b) TP0→TP1, (c, d) TP0→TP2, and (e, f) TP1→TP2. Each panel shows the ANCOVA coefficient (β₁; ×) with 95% confidence intervals (gray bars) for the association of ΔMMI (left column) or ΔPMI (right column) with the change in each endpoint over the corresponding interval. β₁ represents the expected difference in the endpoint change per 1-percentage-point higher ΔMMI/ΔPMI (i.e., lesser muscle loss or greater gain). The vertical dashed line indicates β₁ = 0. Models were fitted using ordinary least-squares ANCOVA and adjusted for the endpoint value at the start of each interval (TP0 for TP0→TP1 and TP0→TP2; TP1 for TP1→TP2), age, and sex. Increases in BMI, albumin, PNI, and GNRI generally indicate improvement, whereas increases in CRP, GPS, and CAR indicate worsening. ΔMMI/ΔPMI were calculated as (later − earlier) / earlier × 100. Abbreviations: MMI, masseter muscle index; PMI, psoas muscle index; BMI, body mass index; PNI, prognostic nutritional index; GNRI, geriatric nutritional risk index; CRP, C-reactive protein; GPS, Glasgow prognostic score; CAR, C-reactive protein/albumin ratio; TP, time point. Time points: TP0, baseline (pre-CRT); TP1, post-CRT (preoperative); TP2, 3 months postoperatively (±14 days)

**Fig. 4:**
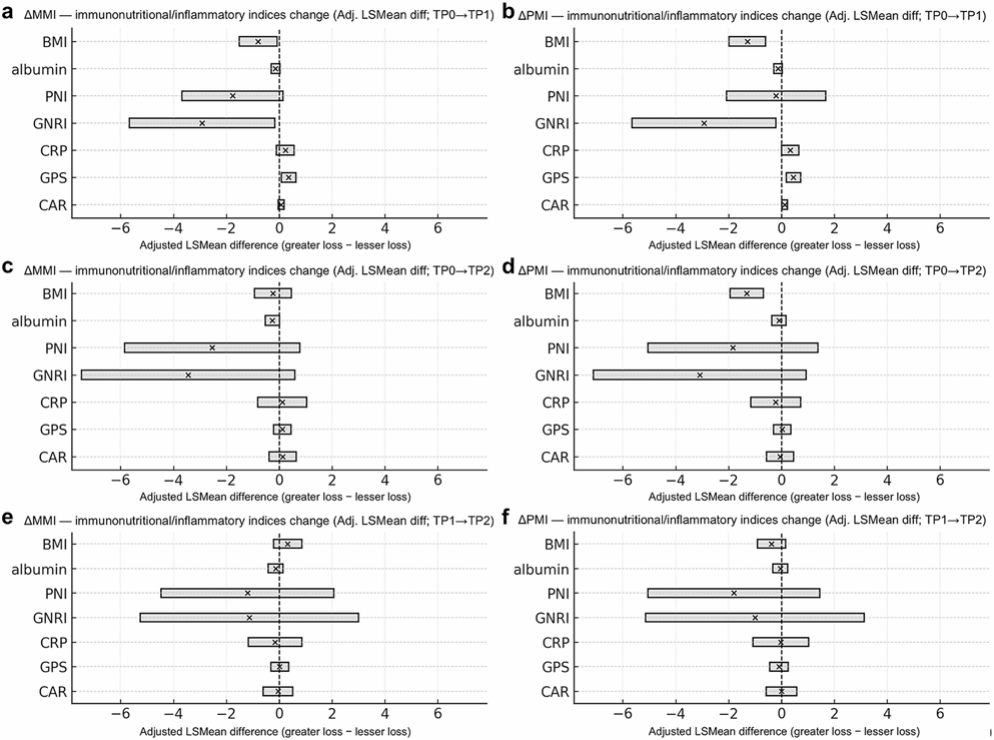
Adjusted LSM differences in immunonutritional/inflammatory indices by early ΔMMI/ΔPMI (median split at TP0→TP1) (a, b) TP0→TP1, (c, d) TP0→TP2, and (e, f) TP1→TP2. Each panel shows the adjusted LSMean difference (×) with 95% confidence intervals (gray bars) for the change in each endpoint, comparing groups defined by a cohort-median split of ΔMMI (left column) or ΔPMI (right column) during TP0→TP1. Differences are expressed as greater loss − lesser loss (x-axis). Groups were defined as greater loss (Δ ≤ median; lower Δ, ties included) and lesser loss (Δ > median; higher Δ). The vertical dashed line indicates 0 (no difference). Models were fitted using ordinary least-squares ANCOVA and adjusted for the endpoint value at the start of each interval (TP0 for TP0→TP1 and TP0→TP2; TP1 for TP1→TP2), age, and sex. Positive values indicate larger increases in the endpoint in the greater-loss group. Increases in BMI, albumin, PNI, and GNRI generally indicate improvement, whereas increases in CRP, GPS, and CAR indicate worsening. Abbreviations: Δ, percent change; MMI, masseter muscle index; PMI, psoas muscle index; BMI, body mass index; PNI, prognostic nutritional index; GNRI, geriatric nutritional risk index; CRP, C-reactive protein; GPS, Glasgow prognostic score; CAR, C-reactive protein/albumin ratio; TP, time point. Time points: TP0, baseline (pre-CRT); TP1, post-CRT (preoperative); TP2, 3 months postoperatively (±14 days)

Figures 3 and 4 show the key adjusted associations for the prespecified core endpoints (BMI, albumin, PNI, GNRI, CRP, GPS, and CAR). Complete numerical results for the adjusted continuous models are provided in Supplementary Table S3, and the corresponding median-split analyses are provided in Supplementary Table S4. Analyses of TP2 endpoints used complete-case data (*n* = 76/78, 97.4%).

#### CRT period (TP0→TP1)

In adjusted continuous models, better preservation of both ΔMMI and ΔPMI was associated with better maintenance of BMI during CRT (ΔMMI: β₁ = +0.0476, *P* = 0.0002; ΔPMI: β₁ = +0.0686, *P* < 0.0001; Supplementary Table S3). Beyond BMI, ΔPMI showed broader favorable associations with immunonutritional status, including higher GNRI and albumin and a lower GPS (GNRI: β₁ = +0.1584, *P* = 0.0090; albumin: β₁ = +0.0074, *P* = 0.0330; GPS: β₁ = −0.0196, *P* = 0.0012), whereas ΔMMI was primarily associated with BMI and a lower GPS (β₁ = −0.0124, *P* = 0.0174). Median-split analyses were directionally consistent (Supplementary Table S4): compared with the lesser-loss groups, the greater-loss groups showed a larger decline in BMI and worse GPS profiles. For ΔPMI stratification, inflammatory markers (CRP and CAR) were also higher in the greater-loss group (Figs. 3 and 4).

#### Postoperative window (TP1→TP2)

Associations between CRT-period muscle changes and postoperative changes were generally attenuated and non-significant, with only limited isolated findings (Supplementary Table S3), including an inverse association between ΔPMI and NLR at TP1→TP2 (β₁ = −0.221, *P* = 0.0496).

#### Combined CRT plus postoperative interval (TP0→TP2)

Over the full interval, ΔPMI remained consistently associated with favorable changes in BMI and major immunonutritional markers (BMI: β₁ = +0.0642, *P* < 0.0001; PNI: β₁ = +0.1685, *P* = 0.0176; GNRI: β₁ = +0.2608, *P* = 0.0034; albumin: β₁ = +0.0122, *P* = 0.0363; Supplementary Table S3). In contrast, ΔMMI showed only borderline associations with BMI and GNRI (BMI: *P* = 0.0760; GNRI: *P* = 0.0535). Similarly, median-split analyses indicated that greater ΔPMI loss was associated with a larger BMI decline across TP0→TP2 (Supplementary Table S4), whereas other endpoints were non-significant or inconsistent.

## Discussion

The findings of this study suggest that the chewing–swallowing (craniofacial) musculature and trunk musculature provide complementary prognostic windows to patients with ESCC undergoing surgery after CRT. In the early treatment interval (TP0→TP1), ΔMMI stratified OS at a borderline-significant level, whereas ΔPMI separated RFS. Notably, an exploratory 2 × 2 stratification combining the median splits of ΔMMI and ΔPMI identified a high-risk “double-loss” phenotype (ΔMMI ≤ median and ΔPMI ≤ median), which tended to show the poorest OS and RFS (Supplementary Fig. S1). This supports a complementary interpretation: considering ΔMMI alongside ΔPMI may enhance risk stratification more than either index alone. In this context, ΔMMI functions as a treatment-sensitive signal reflecting vulnerability of the swallowing apparatus, whereas ΔPMI provides a trunk-oriented systemic window that may track oncologic dynamics more closely. This interpretation aligns with CRT-related toxicities affecting oral intake and with the concept of sarcopenic dysphagia [10]. Consistent with this, a propensity score–matched ESCC study showed that preserved dentition (≥ 7 remaining molars), a surrogate of masticatory capacity, predicted superior OS and RFS independently of postoperative pneumonia [21]. Analytically, Δ-based muscle changes were not stratified by sex; instead, all adjusted analyses included age and sex as covariates.

The biological plausibility is strong. CRT frequently induces mucositis, odynophagia, and reduced intake, promoting selective disuse atrophy of the masseter and related swallowing musculature: a pattern consistent with sarcopenic dysphagia [10]. Conversely, changes in the psoas muscle may reflect global nutritional and inflammatory status (concepts captured by indices such as the PNI and SII); thereby offering a complementary systemic window alongside craniofacial musculature [5, 22, 23]. Consistent with this framework, in our cohort, ΔPMI (TP0→TP1) was consistently associated with deterioration from TP0 to TP2 in BMI and major immunonutritional indices (PNI, GNRI, and albumin), whereas ΔMMI showed weaker or borderline associations. Accordingly, the clinical hypothesis that early maintenance of ΔMMI could help preserve immunonutrition by TP2 is mechanistically reasonable and directionally consistent with our data, yet its statistical support is more limited than that for ΔPMI and should be interpreted cautiously.

Building on this interpretation, the CRT-period loss of the chewing–swallowing musculature could promote dysphagia, limit oral intake, and increase aspiration susceptibility, thereby linking early functional decline with later adverse events such as postoperative pneumonia and delayed recovery. This pathway provides a coherent explanation for the observed patterns, given the established association between postoperative pneumonia and long-term outcomes after esophagectomy [13, 14].

Postoperative pneumonia exerted a profound adverse effect on long-term outcomes; however, early CRT-period muscle changes (ΔMMI/ΔPMI) did not differ remarkably by pneumonia status in this cohort. This pattern suggests that postoperative pneumonia acts, at least in part, independently of tumor biology to worsen long-term outcomes [13, 14]. Accordingly, programs incorporating oral care, swallowing rehabilitation, and nutrition support are reasonable components of perioperative pathways to mitigate pulmonary complications [24, 25].

Several methodological cautions apply. First, percent change is sensitive to baseline scale and regression to the mean; therefore, we reported absolute values at TP0 and TP1 together with percent changes for muscle indices, and adjusted absolute changes for endpoints, to reduce over-interpretation. Second, survival contrasts were performed using median splits as a simple and reproducible stratification, and borderline P-values should be interpreted conservatively. Defining clinically meaningful ΔMMI thresholds will require larger cohorts, external validation, and time-to-event methods that handle censoring appropriately.

Future work should validate these findings prospectively and assess whether continuous ΔMMI/ΔPMI, combined with simple clinical and laboratory features, can yield a pragmatic perioperative risk tool. Joint longitudinal–survival or mediation frameworks could clarify whether postoperative complications, particularly pneumonia, lie on the causal pathway from early treatment-period muscle loss to adverse survival. Perioperative oral management is associated with fewer postoperative pneumonias after esophagectomy consistently, supporting the inclusion of dental and oral care within risk-mitigation pathways [24–26]. Beyond oral care, multimodal prehabilitation combining exercise and nutrition has also been associated with fewer complications, particularly pulmonary events, and improved recovery before and after esophagectomy [27, 28].

## Limitations

This single-center, retrospective study with a small sample size limits precision and generalizability and may entail selection bias. Importantly, the cohort was limited to patients who underwent esophagectomy after CRT; thus, the findings may not be generalizable to patients treated with definitive CRT alone without surgery. CT-based muscle indices were available only at TP0 (pre-CRT) and TP1 (post-CRT, pre-surgery), post-TP1 trajectories were not captured, and small differences in acquisition or slice selection may introduce measurement variability. Moreover, measurements were taken by a single evaluator, and we did not conduct a formal intra-/interobserver reproducibility assessment, such as ICC, within our dataset. However, a prior CT-based study reported high intra- and interobserver reliability for masseter muscle area measurements when standardized landmarks are applied (for example, 96.3% intraobserver and 96.7% interobserver reliability) [29]. Survival contrasts were exploratory median-split analyses without multiplicity adjustment, so residual confounding such as stage, treatment tolerance, and comorbidities may remain. Although we explored an ROC-derived ΔMMI cut-off as a sensitivity analysis, standard ROC does not account for censoring or time-dependence; therefore, clinically useful ΔMMI thresholds should be established in larger cohorts using time-to-event methods appropriate for censored outcomes. Swallowing assessments were limited (no videofluoroscopy/FEES, tongue pressure, or bite force), and dental measures were outside the scope of the present analysis. Moreover, external validation was not performed and the predominantly male sample limited sex-stratified analyses; therefore, Δ-based muscle changes were analyzed as pooled data with adjustment for age and sex. However, residual sex-specific effects cannot be ruled out. Future multicenter prospective studies should standardize CT protocols, integrate objective swallowing and dental metrics, validate prognostic models externally, and evaluate MMI-guided prehabilitation.

## Conclusion

In patients with ESCC undergoing esophagectomy after CRT, early masseter decline (ΔMMI TP0→TP1) was associated with marginally worse OS, whereas early psoas decline (ΔPMI TP0→TP1) was associated with worse RFS. Across TP0→TP2, ΔPMI tracked immunonutritional deterioration (BMI, PNI, GNRI, albumin) more consistently than ΔMMI, whereas postoperative pneumonia showed a significant, largely independent adverse association with survival. These complementary signals support integrating muscle imaging (MMI/PMI) with routine immunonutritional assessment and proactive perioperative support. Thus, maintaining ΔMMI early may help mitigate immunonutritional decline by TP2, through the preservation of swallowing function and oral intake; however, the statistical evidence is limited, and prospective confirmation is warranted.

## Electronic Supplementary Material

Below is the link to the electronic supplementary material.

Supplementary Material 1

Supplementary Material 2

Supplementary Material 3

Supplementary Material 4

Supplementary Material 5: Kaplan–Meier survival curves by combined stratification of early ΔMMI and ΔPMI (TP0→TP1). (a) Overall survival (OS) and (b) recurrence-free survival (RFS) according to a 2×2 classification usingcohort-median splits of ΔMMI and ΔPMI. Patients were categorized into four groups: ΔPMI > median and ΔMMI > median (n = 22), ΔPMI ≤ median and ΔMMI > median (n = 17), ΔPMI > median and ΔMMI ≤ median (n = 17), and ΔPMI ≤ median and ΔMMI ≤ median (n = 22). Percent change (Δ) was calculated as (TP1 − TP0) / TP0 × 100; median cutoffs were −6.98% for ΔMMI and −4.70% for ΔPMI. Tick marks indicate censoring, and numbers at risk are shown below each plot. Curves were compared using the log-rank test (P values shown). Abbreviations: MMI, masseter muscle index; PMI, psoas muscle index; OS, overall survival; RFS, recurrence-free survival; TP0, baseline (pre-CRT); TP1, post-CRT (preoperative)
