# Supplementary material for: Masseter muscle change during chemoradiotherapy for esophageal cancer: survival and immunonutritional associations compared with the psoas muscle index
Source: Surg Today. 2026 Apr 24;56(9):1881–90. doi: 10.1007/s00595-026-03288-y (PMC13473311; doi:10.1007/s00595-026-03288-y)
Supplement: Supplementary file 1 [file 595_2026_3288_MOESM1_ESM.docx]

**Supplementary Table S1. Sensitivity Cox model for overall survival (n=78)**

| **Covariate** | **HR** | **95% CI** | **P** |
| --- | --- | --- | --- |
| Lesser-loss ΔMMI group (ΔMMI > median) | 0.4912 | 0.2529–0.9244 | 0.0307 |
| Mellow–Pinkas dysphagia score (per 1 grade) | 0.8355 | 0.6141–1.1265 | 0.2441 |
| Tumor location: Cervical/Upper vs Middle/Lower | 1.2916 | 0.5595–2.7219 | 0.5215 |

Cox proportional hazards model for overall survival including ΔMMI strata and additionally adjusting for baseline Mellow–Pinkas dysphagia score (TP0) and dichotomized tumor location (Cervical/Upper vs Middle/Lower). The greater-loss ΔMMI group (ΔMMI ≤ cohort median over TP0→TP1) served as the reference. Abbreviations: CI, confidence interval; HR, hazard ratio; MMI, masseter muscle index.
