# Supplementary material for: Masseter muscle change during chemoradiotherapy for esophageal cancer: survival and immunonutritional associations compared with the psoas muscle index
Source: Surg Today. 2026 Apr 24;56(9):1881–90. doi: 10.1007/s00595-026-03288-y (PMC13473311; doi:10.1007/s00595-026-03288-y)
Supplement: Supplementary file 2 [file 595_2026_3288_MOESM2_ESM.docx]

**Supplementary Table S2. Additional immunonutritional and immune–inflammation indices (platelet count, CONUT, NLR, PLR, LMR, and SII) stratified by ΔMMI (TP0→TP1) median**

| **Variable** | **All patients** | **ΔMMI Greater Loss (≤ median) n=39** | **ΔMMI Lesser Loss (> median) n=39** | **P value** |
| --- | --- | --- | --- | --- |
| **TP0 (baseline) (n=78)** |  |  |  |  |
| Platelet (10^3^/μL) | 266 (224-321) | 259 (204-312) | 279 (246-323) | 0.2265 |
| CONUT  0/1/2/3/4/5/6  /7/8/9/10/11/12 | 25/29/14/5/1/1/1  /0/1/1/0/0/0 | 14/17/6/2/0/0/0  /0/0/0/0/0/0 | 11/12/8/3/1/1/1  /0/1/1/0/0/0 | 0.5685 |
| NLR | 2.87 (1.90-4.34) | 3.60 (1.90-4.57) | 2.40 (1.90-3.21) | 0.0838 |
| PLR | 153.9 (117.9-202.2) | 157.5 (118.3-208.5) | 149.8 (114.6-191.1) | 0.4780 |
| LMR | 4.07 (3.02-5.58) | 3.91 (2.84-5.99) | 4.30 (3.36-5.30) | 0.8730 |
| SII | 741.8 (469.6-1133.0) | 901.3 (447.2-1227.6) | 679.1 (526.4-1075.6) | 0.4780 |
| **TP1 (post-CRT, preoperative) (n=78)** |  | **ΔMMI Greater Loss (≤ median) n=39** | **ΔMMI Lesser Loss (> median) n=39** | **P value** |
| Platelet (10^3^/μL) | 203 (146-244) | 201 (148-237) | 207 (152-254) | 0.3928 |
| CONUT  0/1/2/3/4/5/6  /7/8/9/10/11/12 | 1/3/19/23/12/10/2  /4/3/1/0/0/0 | 1/1/10/12/5/6/0  /2/1/1/0/0/0 | 0/2/9/11/7/4/2  /2/2/0/0/0/0 | 0.7891 |
| NLR | 3.68 (2.21-4.85) | 3.91 (2.69-5.47) | 2.87 (2.04-4.34) | 0.0265 |
| PLR | 221.0 (162.9-343.8) | 239.5 (166.3-399.8) | 220.4 (154.5-306.4) | 0.3274 |
| LMR | 2.18 (1.64-3.12) | 2.03 (1.40-2.73) | 2.46 (1.80-3.43) | 0.0803 |
| SII | 671.5 (402.7-1138.4) | 791.0 (527.6-1243.6) | 542.2 (398.4-980.9) | 0.1238 |
| **TP2 (3 months postoperatively) (n=76)** |  | **ΔMMI Greater Loss (≤ median) n=38** | **ΔMMI Lesser Loss (> median) n=38** | **P value** |
| Platelet (10^3^/μL) | 226 (173-276) | 217 (160-275) | 235 (184-277) | 0.3339 |
| CONUT  0/1/2/3/4/5/6  /7/8/9/10/11/12 | 0/5/15/20/11/5/5  /2/2/4/4/2/1 | 0/2/9/10/5/0/4  /0/2/2/2/1/1 | 0/3/6/10/6/5/1  /2/0/2/2/1/0 | 0.3140 |
| NLR | 4.47 (2.39-7.54) | 5.28 (2.39-7.78) | 4.21 (2.39-7.38) | 0.6334 |
| PLR | 288.1 (165.3-476.7) | 288.1 (164.1-466.2) | 280.9 (188.5-487.7) | 0.7465 |
| LMR | 2.17 (1.45-3.70) | 2.12 (1.25-3.46) | 2.29 (1.49-3.72) | 0.4392 |
| SII | 1065.4 (445.2-1912.8) | 1035.1 (416.4-1962.4) | 1133.9 (481.3-1837.1) | 0.7305 |

Data are presented as median (interquartile range [IQR]) or n values, as appropriate. P values compare ΔMMI strata (ΔMMI ≤ cohort median vs. ΔMMI > cohort median) at each time point and were calculated using the Mann–Whitney U test for continuous variables (two-sided) and Pearson’s chi-square test for categorical variables with more than two categories (CONUT score distribution). ΔMMI is the percent change in MMI during CRT (TP0→TP1), defined as ([TP1−TP0]/TP0)×100; negative values indicate loss (cohort median ΔMMI = −6.98%). TP0, baseline (pre-CRT); TP1, post-CRT, preoperative assessment; TP2, 3 months postoperatively (±14 days). Denominators may vary because of missing values, as shown. Abbreviations: CONUT, Controlling Nutritional Status; CRT, chemoradiotherapy; LMR, lymphocyte-to-monocyte ratio; MMI, masseter muscle index; NLR, neutrophil-to-lymphocyte ratio; PLR, platelet-to-lymphocyte ratio; SII, systemic immune-inflammation index (platelet × neutrophil / lymphocyte).
