# Supplementary material for: Masseter muscle change during chemoradiotherapy for esophageal cancer: survival and immunonutritional associations compared with the psoas muscle index
Source: Surg Today. 2026 Apr 24;56(9):1881–90. doi: 10.1007/s00595-026-03288-y (PMC13473311; doi:10.1007/s00595-026-03288-y)
Supplement: Supplementary file 3 [file 595_2026_3288_MOESM3_ESM.docx]

Supplementary Table S3. ANCOVA (continuous): adjusted regression coefficients (β₁) for absolute changes in immunonutritional/inflammatory endpoints per 1% increase in ΔMMI% or ΔPMI%

| **Interval** | **Outcome** | **β₁ (ΔMMI)** | **95% CI (ΔMMI)** | **P (ΔMMI)** | **β₁ (ΔPMI)** | **95% CI (ΔPMI)** | **P (ΔPMI)** |
| --- | --- | --- | --- | --- | --- | --- | --- |
| TP0→TP1 |  |  |  |  |  |  |  |
|  | BMI | 0.0476 | [0.0231, 0.0722] | 0.0002 | 0.0686 | [0.0386, 0.0986] | <0.0001 |
|  | albumin | 0.0001 | [-0.0058, 0.0061] | 0.9507 | 0.0074 | [0.0006, 0.0141] | 0.0330 |
|  | PNI | -0.025 | [-0.0946, 0.0445] | 0.4755 | 0.0487 | [-0.0323, 0.1297] | 0.2347 |
|  | GNRI | 0.0418 | [-0.0616, 0.1452] | 0.423 | 0.1584 | [0.0407, 0.2760] | 0.0090 |
|  | CRP | -0.0067 | [-0.0187, 0.0052] | 0.2677 | -0.0144 | [-0.0286, -0.0002] | 0.0460 |
|  | GPS | -0.0124 | [-0.0225, -0.0022] | 0.0174 | -0.0196 | [-0.0313, -0.0080] | 0.0012 |
|  | CAR | -0.0021 | [-0.0061, 0.0019] | 0.2944 | -0.0054 | [-0.0101, -0.0007] | 0.0250 |
|  | platelets | -0.251 | [-1.1506, 0.6485] | 0.5799 | -0.1583 | [-1.2488, 0.9320] | 0.7731 |
|  | CONUT | 0.0187 | [-0.0101, 0.0477] | 0.2001 | -0.0157 | [-0.0499, 0.0184] | 0.3615 |
|  | NLR | 0.0973 | [0.0286, 0.1660] | 0.0061 | 0.0634 | [-0.0205, 0.1473] | 0.1367 |
|  | PLR | 2.354 | [-0.5102, 5.2183] | 0.1057 | 2.8333 | [-0.5659, 6.2327] | 0.1010 |
|  | LMR | -0.0237 | [-0.0430, -0.0044] | 0.0166 | -0.0152 | [-0.0391, 0.0085] | 0.2044 |
|  | SII | 15.6977 | [-2.0448, 33.4402] | 0.082 | 7.6273 | [-13.7910, 29.0458] | 0.4802 |
| TP0→TP2 |  |  |  |  |  |  |  |
|  | BMI | 0.0222 | [-0.0023, 0.0468] | 0.076 | 0.0642 | [0.0357, 0.0928] | <0.0001 |
|  | albumin | 0.0089 | [-0.0006, 0.0186] | 0.068 | 0.0122 | [0.0008, 0.0237] | 0.0363 |
|  | PNI | 0.0572 | [-0.0618, 0.1763] | 0.3413 | 0.1685 | [0.0303, 0.3068] | 0.0176 |
|  | GNRI | 0.1441 | [-0.0022, 0.2905] | 0.0535 | 0.2608 | [0.0893, 0.4324] | 0.0034 |
|  | CRP | -0.0067 | [-0.0399, 0.0263] | 0.6847 | -0.0234 | [-0.0640, 0.0170] | 0.2527 |
|  | GPS | -0.0078 | [-0.0196, 0.0039] | 0.1885 | -0.0146 | [-0.0286, -0.0006] | 0.0405 |
|  | CAR | -0.0035 | [-0.0218, 0.0147] | 0.6989 | -0.0152 | [-0.0374, 0.0069] | 0.1761 |
|  | platelets | -0.7419 | [-1.8106, 0.3267] | 0.1707 | 0.5494 | [-0.8123, 1.9111] | 0.4239 |
|  | CONUT | -0.0225 | [-0.0724, 0.0274] | 0.3722 | -0.0476 | [-0.1043, 0.0090] | 0.0981 |
|  | NLR | 0.0182 | [-0.1649, 0.2014] | 0.8433 | -0.1812 | [-0.3988, 0.0362] | 0.1010 |
|  | PLR | 0.1723 | [-3.6133, 3.9580] | 0.9279 | -1.9584 | [-6.5422, 2.6254] | 0.3971 |
|  | LMR | -0.031 | [-0.0649, 0.0029] | 0.0725 | 0.0141 | [-0.0283, 0.0567] | 0.5079 |
|  | SII | 1.8638 | [-49.8867, 53.6145] | 0.943 | -47.6832 | [-109.9058, 14.5392] | 0.1309 |
| TP1→TP2 |  |  |  |  |  |  |  |
|  | BMI | -0.0105 | [-0.0297, 0.0086] | 0.2777 | 0.0128 | [-0.0105, 0.0362] | 0.2777 |
|  | albumin | 0.0063 | [-0.0037, 0.0163] | 0.2139 | 0.0084 | [-0.0039, 0.0207] | 0.1772 |
|  | PNI | 0.0486 | [-0.0713, 0.1687] | 0.4213 | 0.139 | [-0.0011, 0.2793] | 0.0519 |
|  | GNRI | 0.0822 | [-0.0646, 0.2291] | 0.268 | 0.1284 | [-0.0506, 0.3075] | 0.1572 |
|  | CRP | 0.0011 | [-0.0352, 0.0374] | 0.9512 | -0.0293 | [-0.0747, 0.0161] | 0.2025 |
|  | GPS | -0.0036 | [-0.0155, 0.0081] | 0.5372 | -0.0106 | [-0.0257, 0.0043] | 0.1617 |
|  | CAR | 0.0008 | [-0.0192, 0.0209] | 0.9344 | -0.0164 | [-0.0417, 0.0087] | 0.1975 |
|  | platelets | -0.5959 | [-1.6144, 0.4225] | 0.2473 | 0.4984 | [-0.7778, 1.7747] | 0.4388 |
|  | CONUT | -0.0125 | [-0.0633, 0.0383] | 0.6253 | -0.0417 | [-0.0993, 0.0158] | 0.1525 |
|  | NLR | 0.0032 | [-0.1905, 0.1970] | 0.9736 | -0.221 | [-0.4417, -0.0003] | 0.0496 |
|  | PLR | -0.4448 | [-4.2818, 3.3921] | 0.8178 | -3.158 | [-7.7879, 1.4718] | 0.1781 |
|  | LMR | -0.0182 | [-0.0542, 0.0177] | 0.316 | 0.0243 | [-0.0186, 0.0673] | 0.2630 |
|  | SII | -4.4816 | [-56.4944, 47.5312] | 0.8641 | -54.0977 | [-114.9713, 6.7759] | 0.0807 |

Outcome changes were analyzed as absolute changes (ΔZ = Z_end − Z_start) over each interval. Separate ANCOVA models were fitted with ΔMMI% or ΔPMI% as the predictor (to avoid collinearity). Models were adjusted for the outcome’s starting value at the beginning of the interval (TP0 for TP0→TP1 and TP0→TP2; TP1 for TP1→TP2), age (years at TP0), and sex. β₁ denotes the adjusted slope in ΔZ per 1% increase in ΔMMI% or ΔPMI%, reported with 95% confidence intervals (CIs) and two-sided P values. CONUT was analyzed as a continuous numeric variable. TP0, baseline (pre-CRT); TP1, post-CRT, preoperative assessment; TP2, 3 months postoperatively (±14 days). Abbreviations: BMI, body mass index; CAR, C-reactive protein/albumin ratio; CONUT, controlling nutritional status; CRP, C-reactive protein; CRT, chemoradiotherapy; GNRI, geriatric nutritional risk index; GPS, Glasgow Prognostic Score; LMR, lymphocyte-to-monocyte ratio; MMI, masseter muscle index; NLR, neutrophil-to-lymphocyte ratio; PLR, platelet-to-lymphocyte ratio; PMI, psoas muscle index; PNI, prognostic nutritional index; SII, systemic immune-inflammation index (platelet × neutrophil / lymphocyte).
