# Supplementary material for: Masseter muscle change during chemoradiotherapy for esophageal cancer: survival and immunonutritional associations compared with the psoas muscle index
Source: Surg Today. 2026 Apr 24;56(9):1881–90. doi: 10.1007/s00595-026-03288-y (PMC13473311; doi:10.1007/s00595-026-03288-y)
Supplement: Supplementary file 4 [file 595_2026_3288_MOESM4_ESM.docx]

Supplementary Table S4. ANCOVA (median split): adjusted LSMean differences in immunonutritional/inflammatory endpoints by ΔMMI/ΔPMI group

| **Interval** | **Outcome** | **Adj. LSMean diff(ΔMMI)** | **95%CI (ΔMMI group)** | **p (ΔMMI group)** | **Adj. LSMean diff (ΔPMI)** | **95%CI (ΔPMI group)** | **p (ΔPMI group)** |
| --- | --- | --- | --- | --- | --- | --- | --- |
| **TP0→TP1** |  |  |  |  |  |  |  |
|  | BMI | -0.795 | [-1.5099, -0.0802] | 0.0298 | -1.2914 | [-1.9808, -0.6021] | 0.0004 |
|  | albumin | -0.1388 | [-0.3008, 0.0231] | 0.092 | -0.1254 | [-0.2849, 0.0340] | 0.1212 |
|  | PNI | -1.7626 | [-3.6761, 0.1508] | 0.0704 | -0.2037 | [-2.0799, 1.6724] | 0.8292 |
|  | GNRI | -2.9147 | [-5.6605, -0.1689] | 0.0378 | -2.9297 | [-5.6521, -0.2073] | 0.0353 |
|  | CRP | 0.2349 | [-0.0976, 0.5675] | 0.1634 | 0.3332 | [0.0007, 0.6657] | 0.0495 |
|  | GPS | 0.3536 | [0.0755, 0.6318] | 0.0134 | 0.4537 | [0.1827, 0.7248] | 0.0013 |
|  | CAR | 0.0711 | [-0.0413, 0.1835] | 0.2115 | 0.1209 | [0.0104, 0.2313] | 0.0324 |
|  | platelets | 6.6318 | [-18.3969, 31.6607] | 0.5991 | -1.2276 | [-26.6568, 24.2015] | 0.9236 |
|  | CONUT | 0.2305 | [-0.5914, 1.0525] | 0.5779 | -0.0843 | [-0.8770, 0.7084] | 0.8328 |
|  | NLR | -0.8779 | [-2.9056, 1.1497] | 0.3911 | -1.2681 | [-3.2332, 0.6968] | 0.2025 |
|  | PLR | 7.6702 | [-73.2743, 88.6148] | 0.8507 | -64.0524 | [-142.7485, 14.6435] | 0.1091 |
|  | LMR | 0.3741 | [-0.1761, 0.9244] | 0.1796 | 0.4795 | [-0.0659, 1.0251] | 0.084 |
|  | SII | -28.6097 | [-532.9201, 475.7006] | 0.9103 | -180.3403 | [-681.2497, 320.5691] | 0.4754 |
| **TP0→TP2** |  |  |  |  |  |  |  |
|  | BMI | -0.2317 | [-0.9248, 0.4613] | 0.5072 | -1.3114 | [-1.9467, -0.6760] | 0.0001 |
|  | albumin | -0.2564 | [-0.5234, 0.0105] | 0.0595 | -0.0909 | [-0.3594, 0.1774] | 0.5013 |
|  | PNI | -2.5332 | [-5.8490, 0.7825] | 0.1321 | -1.8363 | [-5.0496, 1.3769] | 0.2583 |
|  | GNRI | -3.4417 | [-7.4776, 0.5940] | 0.0934 | -3.0837 | [-7.1074, 0.9399] | 0.1309 |
|  | CRP | 0.1165 | [-0.8112, 1.0443] | 0.803 | -0.2163 | [-1.1550, 0.7224] | 0.6474 |
|  | GPS | 0.1215 | [-0.2062, 0.4493] | 0.4622 | 0.0315 | [-0.2983, 0.3613] | 0.8495 |
|  | CAR | 0.1304 | [-0.3813, 0.6421] | 0.613 | -0.0462 | [-0.5603, 0.4678] | 0.8582 |
|  | platelets | 10.0894 | [-20.2639, 40.4428] | 0.5097 | -14.414 | [-45.2614, 16.4333] | 0.3547 |
|  | CONUT | 0.6923 | [-0.6760, 2.0606] | 0.3164 | 0.4217 | [-0.8981, 1.7415] | 0.526 |
|  | NLR | 1.5875 | [-3.6241, 6.7992] | 0.5455 | 2.6099 | [-2.4641, 7.6840] | 0.3085 |
|  | PLR | 44.1511 | [-61.8216, 150.1239] | 0.4089 | 19.2571 | [-86.2135, 124.7278] | 0.7169 |
|  | LMR | 0.588 | [-0.3768, 1.5530] | 0.2283 | -0.2539 | [-1.2260, 0.7180] | 0.604 |
|  | SII | 494.8419 | [-961.6883, 1951.3722] | 0.5003 | 663.9495 | [-787.9632, 2115.8622] | 0.3649 |
| **TP1→TP2** |  |  |  |  |  |  |  |
|  | BMI | 0.3213 | [-0.2120, 0.8546] | 0.2337 | -0.3736 | [-0.9042, 0.1569] | 0.1646 |
|  | albumin | -0.1325 | [-0.4123, 0.1472] | 0.3481 | -0.0429 | [-0.3269, 0.2410] | 0.764 |
|  | PNI | -1.1956 | [-4.4640, 2.0726] | 0.4681 | -1.8011 | [-5.0511, 1.4487] | 0.2729 |
|  | GNRI | -1.1271 | [-5.2527, 2.9984] | 0.5877 | -1.002 | [-5.1416, 3.1375] | 0.6309 |
|  | CRP | -0.1506 | [-1.1648, 0.8634] | 0.7679 | -0.0175 | [-1.0686, 1.0335] | 0.9735 |
|  | GPS | 0.0175 | [-0.3174, 0.3525] | 0.9171 | -0.0911 | [-0.4397, 0.2574] | 0.6038 |
|  | CAR | -0.04 | [-0.6012, 0.5210] | 0.8872 | 0.0026 | [-0.5797, 0.5850] | 0.9929 |
|  | platelets | 7.86 | [-20.9925, 36.7126] | 0.5888 | -10.3999 | [-39.2248, 18.4249] | 0.4743 |
|  | CONUT | 0.3518 | [-0.9954, 1.6991] | 0.6041 | 0.3764 | [-0.9684, 1.7213] | 0.5784 |
|  | NLR | 1.0501 | [-4.1181, 6.2185] | 0.6866 | 3.5678 | [-1.5260, 8.6617] | 0.1669 |
|  | PLR | 32.1774 | [-72.5692, 136.9241] | 0.5421 | 55.6222 | [-49.7880, 161.0325] | 0.2963 |
|  | LMR | 0.3236 | [-0.6642, 1.3115] | 0.5157 | -0.6465 | [-1.6357, 0.3425] | 0.1966 |
|  | SII | 433.8992 | [-987.7640, 1855.5624] | 0.5447 | 830.4187 | [-580.7348, 2241.5723] | 0.2445 |

Adjusted least-squares mean (LSMean) differences (greater loss − lesser loss) with 95% confidence intervals (CIs) and two-sided P values were estimated using ANCOVA models for absolute outcome changes (ΔZ = Z_end − Z_start), adjusted for the interval’s starting value of each endpoint (Z_start), age (years at TP0), and sex. Intervals denote change windows (TP0→TP1, TP0→TP2, TP1→TP2). ΔMMI and ΔPMI groups were defined by the cohort median percent change over TP0→TP1 (greater loss: Δ ≤ median; lesser loss: Δ > median), and the same grouping defined during CRT (TP0→TP1) was applied to all intervals shown. TP0, baseline (pre-CRT); TP1, post-CRT, preoperative assessment; TP2, 3 months postoperatively (±14 days). Abbreviations: Adj. LSMean diff, adjusted least-squares mean difference; BMI, body mass index; CAR, C-reactive protein/albumin ratio; CONUT, controlling nutritional status; CRP, C-reactive protein; CRT, chemoradiotherapy; GNRI, geriatric nutritional risk index; GPS, Glasgow Prognostic Score; LMR, lymphocyte-to-monocyte ratio; MMI, masseter muscle index; NLR, neutrophil-to-lymphocyte ratio; PLR, platelet-to-lymphocyte ratio; PMI, psoas muscle index; PNI, prognostic nutritional index; SII, systemic immune-inflammation index (platelet × neutrophil / lymphocyte).
